# Proceedings of the Medical Society of the City of Galesburg, Held Sept. 7th, 1860

**Published:** 1860-10

**Authors:** M. K. Taylor, H. M. Starkloff

**Affiliations:** President; Secretary


### PROCEEDINGS OF THE MEDICAL SOCIETY OF THE CITY OF GALESBURG, HELD SEPT. 8th, 1860.

At a regular meeting of the Galesburg City Medical Society, held Sept. 8th, 1860, the following report of a committee oppointed at the previous session, was received and adopted unanimously, and copies ordered to be furnished to the *Chicago Medical Journal*, and *Chicago Medical Examiner*, for publication:

REPORT.

Your Committee to whom were referred the several subjects relating to the necessities for some legislation favoring the study of *Practical* Anatomy by Students and members of the Medical Profession; for a Registration Law of Births, Marriages and Deaths, and for the extension to medical men of the privilege of withholding, when called as witnesses, any information derived in the exercise of their professional duties, and which was necessary to the proper discharge thereof; submit the following preamble and resolutions as expressive of the sense of this Society:

*Whereas*, in the study of the science of Medicine and Surgery, and to the proper understanding and practice of the same, it is indispensably necessary that the student have an opportunity to become familiar with the science of *Human Anatomy* by practical dissections with his own hand; and whereas, such course of study is held as pre-requsite to graduation in all well regulated medical schools, and as also held by the public to be of paramount importance to a thorough medical education; and whereas, both the law and the public hold medical men pecuniarly and morally responsible for knowledge of this science in the exercise of their vocation; and whereas, the study of Human Anatomy cannot be pursued in this state except in direct violation of the laws thereof, and the liability to severe fines and penalties; and whereas, we believe this inconsistency and injustice of the law in the requirement of the exercise of this knowledge under penalty, which at the same time, under like penalty, it prohibits being obtained—should be removed; and whereas, we believe that a proper registration of births, marriages and deaths, is necessary in a legal, medical and sanitary point of view, and will conduce greatly to the general welfare of the inhabitants and the advancement of the industrial interests of the State;—

And whereas, by the common law of the land and by the rulings of the courts in this State, physicians and surgeons may be made to disclose any information acquired confidentially in their attendance professionally upon any person, no matter how repugnant to their sense of propriety, honor and morality, such may be;—therefore,

*Resolved*, That we deem it wise and necessary on the part of the Legislature of this State to make some provision which will admit students of medicine and members of the profession to supply themselves with "*material*" from subjects interred at the public expense, when not claimed by relatives or friends, under such restrictions for the protection of the public interest and feelings as the delicate nature of the case may suggest.

*Resolved*, That a law for the registration of births, marriages and deaths, will contribute much to a better understanding of our climate, vital statistics, sanitary and industrial condition; to say nothing of the legal, historical and medical knowledge otherwise accruing therefrom.

*Resolved*, That we hold the relation of physician and patient to be of the most private, personal and confidential character; and as such should ever be held inviolate, and as exempt from inquisitorial proceedings as those of the attorney and his client; and in accordance with these sentiments we claim " that no person duly authorised to practise medicine and surgery should be compelled to disclose any information which he may have acquired in attendance on any patient in a professional character, and which information was *necessary* to enable him to prescribe intelligently as a physician, or do any act as a surgeon."

*Resolved*, That the statutes of the States of New York, Michigan, Iowa, Wisconsin and Missouri, extending to the medical profession these legal privileges, are founded in justice and good morals, and have a proper regard for the peace of families and the community; and therefore, receive our entire approval, in meeting the wants of the profession in this State.

*Resolved*, That at the proper time this Society will propose a memorial to the Legislature, calling attention to these, as we believe, rightfully statutory provisions, and ask for such enactments as the several cases herewith mentioned may require.

*Resolved*, That we recommend similar action to other medical societies, and solicit their active co-operation in carrying out the spirit of these resolutions.

M. K. TAYLOR, M. D.,
*President.*
H. M. STARKLOFF, M. D.,
*Secretary.*

